# Gender-based violence during COVID-19 outbreak in Spain

**DOI:** 10.1017/S0033291720005024

**Published:** 2020-12-07

**Authors:** Roberto Rodriguez-Jimenez, Natalia E. Fares-Otero, Lorena García-Fernández

**Affiliations:** 1 Department of Psychiatry, Instituto de Investigación Sanitaria Hospital 12 de Octubre (imas12), Av. Córdoba s/n, 28041, Madrid, Spain; 2 CIBERSAM (Biomedical Research Networking Centre in Mental Health), Spain; 3 Facultad de Medicina, Universidad Complutense de Madrid (UCM), Plaza Ramón y Cajal, s/n, 28040 Madrid, Spain; 4 Clinical Medicine Department, Universidad Miguel Hernández, Ctra. de Valencia, Km 87, 03550 San Juan, Alicante, Spain; 5 Department of Psychiatry, Hospital Universitario de San Juan, Ctra, N-332, s/n, 03550 San Juan, Alicante, Spain

**Keywords:** Confinement, COVID-19, gender-based violence

In March 2020, the World Health Organization declared coronavirus disease 2019 (COVID-19) outbreak a health emergency of international concern and consequently, government entities in different countries, including Spain, took drastic measures to restrict the mobility of its inhabitants (Real Decreto 463/2020, 2020). One of the most drastic measures carried out in Spain has been the home confinement of the whole population in force from March 14 to May 1. Subsequently, the measures to mitigate confinement were started gradually until June 21 when previous normality was reached.

Domestic violence, especially gender-based violence (GBV), is a main problem that has just gained the health and social importance it deserves a few decades ago. Different legislative and social sensitization measures have been developed to try to alleviate this problem. The pandemic situation caused by severe acute respiratory syndrome-coronavirus-2 (SARS-COV-2) and, especially, home confinement measures have favored the coincidence of a series of factors that may have precipitated or worsened situations of GBV. On the one hand, the initial fear of an unknown pandemic situation, which has exposed the population to a new potentially fatal virus, has been associated with high levels of anxiety, depressive symptoms and symptoms of acute stress that our group has already published (García-Fernández et al., 2020; García-Fernández, Romero-Ferreiro, López-Roldán, Padilla, & Rodriguez-Jimenez, 2020). Moreover, the economic and employment problems resulting from the pandemic have undoubtedly contributed to increasing stressful situations within the family. And finally, home confinement requires a forced and prolonged coexistence of aggressors and victims. Although some studies have already been published warning about the possible negative influence of the pandemic situation on GBV, there is a great scarcity of published data addressing this topic (Barbara et al., 2020; Sediri et al., 2020).

Spain has had for years a specific helpline to request help and advice in situations of GBV. By dialing 016, women who are victims of domestic violence are cared for by specialized personnel without leaving any trace.

**Fig. 1.**
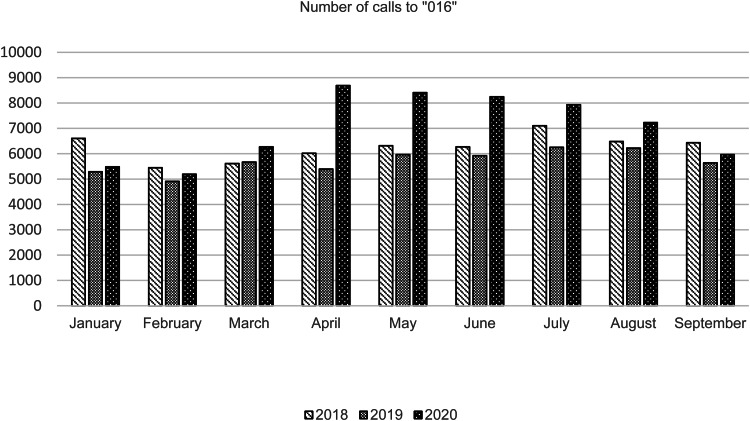
GBV helpline calls. Data obtained from the website of the Ministry of Equality of Spain (Ministry of Equality, 2020).

To the best of our knowledge, there are no published studies addressing the GBV impact of the COVID-19 outbreak in Spain. For this reason, we have recorded the volume of calls to 016 that have occurred during the months of home confinement and we have compared it with the calls received during the same period of the previous 2 years. The following figure shows the data obtained from the website of the Ministry of Equality of Spain (Ministerio de igualdad, 2020) (Fig. 1).

As the figure shows, in March (home confinement started on the 14th) there is already a slight increase in the number of calls asking for help due to GBV. However, in April, May and June this increase seems evident when compared to the number of calls registered in the previous 2 years. In August, the number of calls seem to start declining to equalize in September to those made in the last two previous years.

This cross-sectional analysis does not allow to establish a causality association between home confinement and the higher number of calls requesting help due to GBV, but the relationship between the beginning of the home confinement in Spain and the increase in helpline calls seems evident. This increase has persisted through July, when there were no longer any home confinement measures in force (they finished in June 21st) and a decrease in the number of calls began in August, reaching a similar load to previous years by September (October data were not available when this correspondence was written).

The pandemic situation caused by SARS-COV-2 and especially, home confinement, pose risk factors for GBV. We have already warned about the need for clinical screening in vulnerable populations (Fares-Otero, Pfaltz, Estrada-Lorenzo, & Rodriguez-Jimenez, 2020), and the data presented here should alert us to the urgent need to carry out studies on GBV during the COVID-19 pandemic, and to add support measures to the existing ones to treat and to prevent the increase in gender violence that the current pandemic situation may bring, precisely now that we are immersed in the second wave.
